# Vascular contributions to the neurobiological effects of prenatal alcohol exposure

**DOI:** 10.3389/adar.2023.10924

**Published:** 2023-04-12

**Authors:** Sarah Z. Momin, Jacqueline T. Le, Rajesh C. Miranda

**Affiliations:** School of Medicine, Texas A&M University, Bryan, TX, United States

**Keywords:** hypertension, fetal alcohol spectrum disorder, cardiovascular system, vascular pathology, retina

## Abstract

**Background:** Fetal alcohol spectrum disorders (FASD) are often characterized as a cluster of brain-based disabilities. Though cardiovascular effects of prenatal alcohol exposure (PAE) have been documented, the vascular deficits due to PAE are less understood, but may contribute substantially to the severity of neurobehavioral presentation and health outcomes in persons with FASD.

**Methods:** We conducted a systematic review of research articles curated in PubMed to assess the strength of the research on vascular effects of PAE. 40 pertinent papers were selected, covering studies in both human populations and animal models.

**Results:** Studies in human populations identified cardiac defects, and defects in vasculature, including increased tortuosity, defects in basement membranes, capillary basal hyperplasia, endarteritis, and disorganized and diminished cerebral vasculature due to PAE. Preclinical studies showed that PAE rapidly and persistently results in vasodilation of large afferent cerebral arteries, but to vasoconstriction of smaller cerebral arteries and microvasculature. Moreover, PAE continues to affect cerebral blood flow into middle-age. Human and animal studies also indicate that ocular vascular parameters may have diagnostic and predictive value. A number of intervening mechanisms were identified, including increased autophagy, inflammation and deficits in mitochondria. Studies in animals identified persistent changes in blood flow and vascular density associated with endocannabinoid, prostacyclin and nitric oxide signaling, as well as calcium mobilization.

**Conclusion:** Although the brain has been a particular focus of studies on PAE, the cardiovascular system is equally affected. Studies in human populations, though constrained by small sample sizes, did link pathology in major blood vessels and tissue vasculature, including brain vasculature, to PAE. Animal studies highlighted molecular mechanisms that may be useful therapeutic targets. Collectively, these studies suggest that vascular pathology is a possible contributing factor to neurobehavioral and health problems across a lifespan in persons with a diagnosis of FASD. Furthermore, ocular vasculature may serve as a biomarker for neurovascular health in FASD.

## Introduction

Prenatal alcohol exposure (PAE) is well documented to result in a range of adverse physical and neurobehavioral outcomes that are collectively subsumed under the term *Fetal alcohol spectrum disorders* (FASD). Though not by itself an accepted diagnostic term, FASD is a comprehensive umbrella term that includes several diagnostic classifications (1, 2). At the severe end of the FASD continuum, affected individuals may exhibit musculoskeletal and growth deficits, and experience profound craniofacial anomalies, including ocular defects, mid-face hypoplasia and/or cleft palate, profound brain anomalies such as agenesis of the corpus callosum, microencephaly, and intellectual disability (3-8). Evidence for a cluster of presenting symptoms, including mid-face anomalies, growth deficiencies including brain growth deficits and neirobehavioral impairment allows for the diagnosis of *Fetal Alcohol Syndrome* [FAS, for a comprehensive description of diagnostic criteria, see (2)]. However, a majority of persons along the FASD continuum do not exhibit obvious physical anomalies, but rather neurobehavioral deficits including deficits in memory, attention, mathematical and language skills, and decision making processes (9–11). These deficits can have an equally adverse impact on quality of life. These latter, and more prevalent outcomes, are defined by the term *Neurobehavioral Disorder Associated with Prenatal Alcohol Exposure* (ND-PAE) (12, 13), which was incorporated for the first time into the 5th edition of the Diagnostic and Statistical Manual of American Psychiatric Association as an acceptable diagnosis, absent obvious physical anomalies.

PAE is unfortunately very common. Recent studies have investigated levels of phosphatidyl ethanol in blood samples obtained from newborn infants, a unique molecular adduct formed on erythrocyte phospholipid membranes following ethanol exposure. These studies in Texas (14) and West Virginia (15) in the US, and Ontario in Canada (16) documented positivity for this marker in 8%–15% of newborn samples, indicative of substantial rates of third-trimester exposure in these populations. Even higher rates of positivity have been documented in newborns from selected high-risk populations elsewhere (17). Unsurprisingly, estimates of FASD prevalence are also high. For instance, a recent and large prospective case-ascertainment study in four school systems across the US estimated rates of FASD in school-aged children of 1.1%–5%, with a weighted prevalence estimate of 3.1%–9.8% (18). Worldwide, previous estimates have placed the prevalence of FASD at as high as 11.3% in some regions like South Africa, with rates equivalent to North America in many European nations (19). These data collectively point to the likely outsized contribution of PAE to the burden of developmental disability in North America and world-wide, and emphasize the need to understand the etiology of FASD with the goal of early interventions to mitigate the effects of PAE.

The centrality of brain-based disability to the diagnosis of FASD, and particularly ND/PAE, mean that most research has focused on developmental perturbations to neural cells and on the neurobiology of this disability. However, in this review, we focus on the literature implicating the cardiovascular system, and specifically the contribution of the cerebrovascular system in the etiology of FASD. The cerebrovascular system develops during the peak period of neurogenesis (20), and one recent study suggests that cerebral neurogenesis and angiogenesis is molecularly linked, for example, by a microRNA, miR-9 (21). We previously found miR-9 to be inhibited by ethanol in mouse neural stem cells (22), with inhibition in zebrafish resulting in the loss of brain tissue (23). These data suggest that the effects of ethanol on neurogenesis and angiogenesis are mechanistically linked. Conversely, the loss of the endothelial receptor for endothelin-B (ET_B_) has been documented to result in microencephaly, i.e., reduced brain size (24), a key feature of severe FASD, emphasizing the interdependency of neural and vascular systems in the growth of the fetal brain. Interestingly, a few studies using functional magnetic resonance imaging to document changes in functional network connectivity in the resting state (25) and following functional activation (26, 27), in children and adult persons with a diagnosis of FASD, have assessed changes in the BOLD (Blood Oxygenation Level Dependent) signal. These are an important collection of papers for the purposes of this review, because changes in the BOLD signal equally implicate vascular adaptation and neuronal circuit activation (28), and dysregulation in the BOLD signal may indicate vascular dysfunction. Here we conducted a systematic PubMed review of papers on FASD and the vascular system, selecting papers that documented vascular deficits in both human populations and animal models, as well as papers that tested underlying mechanisms, to identify the current knowledge state and potential knowledge gaps in the field of vascular effects of PAE.

## Methods

**FIGURE 1:**
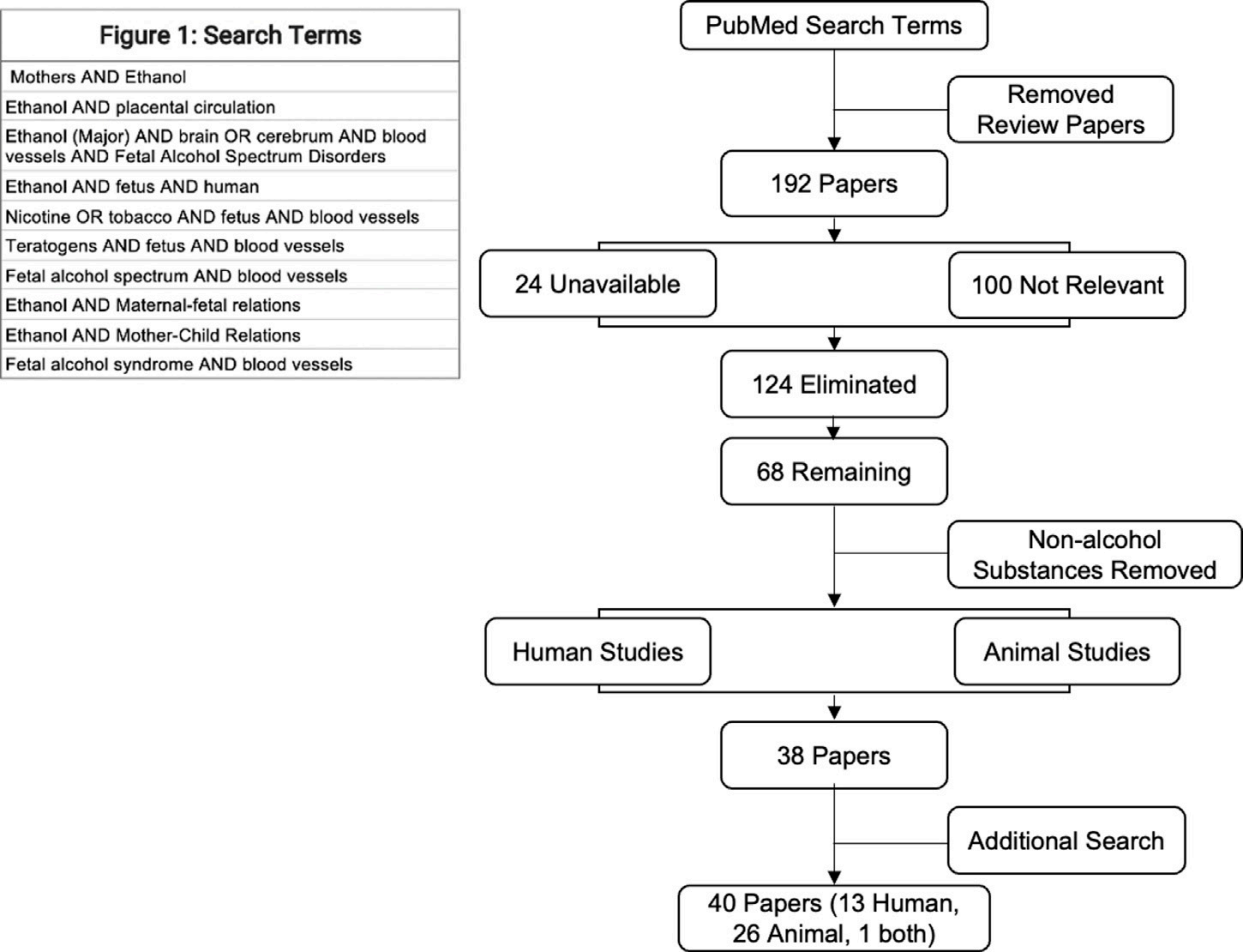
Search terms and schematic for analysis.

We conducted an initial systematic review search in June of 2021 of research articles curated in PubMed to assess the strength of the research on vascular effects of PAE. PubMed was screened for several terms, as listed in Figure 1. After removing all review papers, a total of 191 papers were present using those terms. Of the 191 papers, 24 paper were unavailable to access fully and 99 were unrelated to blood vessels or the cardiovascular system. With 68 papers remaining, non-alcohol related studies were removed, and the remaining 38 studies were then separated by human versus animal models. Subsequent searches conducted until June of 2022 yielded an additional 3 pertinent papers. A total of 40 pertinent papers were selected to be included in this systematic review on the effects of ethanol exposure *in utero* to cardiovascular and neuropsychiatric systems exhibited in both human populations and animal models.

## Results and discussion

### Studies in human populations

**FIGURE 2:**
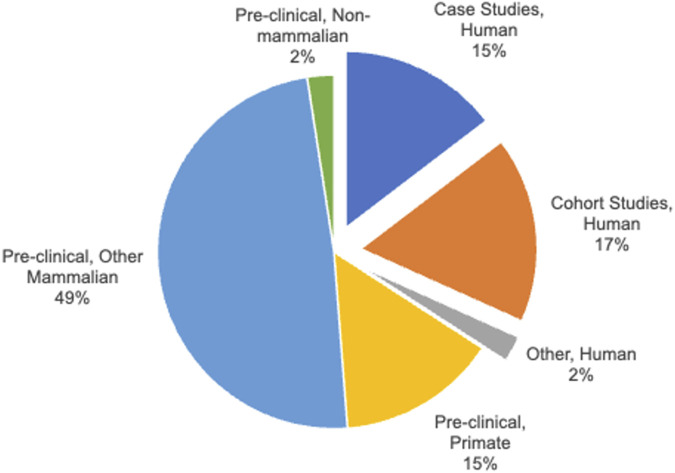
Classification of Human and Pre-clinical animal studies. The category, “Pre-clinical, Other Mammal” includes sheep, rats and mice.

A key, but perhaps unsurprising finding from our analyses is that there are very few studies in human populations, particularly on brain vascular effects of PAE (Figure 2). Many of these studies were case reports, based on very small sample sizes. However, a few studies did include both larger samples of persons with a diagnosis of FAS or pFAS, and importantly, well-described reference or comparison group samples. The earliest description of anatomical anomalies associated with FAS specifically included reference to cardiovascular anomalies, including ventricular septal defects (6, 29), and stenosis of major cardiac efferent arteries like the pulmonary artery (30). Another 1979 case report on two infants with a diagnosis of FAS who were surgically treated for cardiac septal defects (31) also noted that the infants exhibited dysplastic pulmonary arteries, and one infant also exhibited aortic insufficiency. A later case report found evidence for stenosis of the descending aorta and the renal arteries in a child diagnosed with FAS (32). These case reports, despite containing very small patient samples, showed that the heart and large arteries could be affected and perhaps, contribute to the pathology of FAS. Furthermore, vascular deficits within the interstitial vasculature of tissues have also been documented in humans diagnosed with FAS. For instance, in an early report in Lancet by Habbick et al (33), analysis of liver biopsies from three children with FAS uncovered sclerosis and other damage associated with central veins in hepatic lobules and other hepatic vasculature. Additional early supporting data on the vulnerability of interstitial vessels comes from a study in placenta samples obtained from control and alcohol-exposed pregnancies (34). Electron microscopy analysis of placental ultrastructure (five cases from both control and alcohol-exposed pregnancies) found evidence for vascular endarteritis and thickened basal lamina of placental blood vessels, suggestive of a potential inflammatory occlusion of blood vessels and restriction of blood flow. The collective assessment from each of these small-scale studies is that vascular damage may not be limited to the large vessels, but instead may be a general feature of tissues and organs as well. Moreover, such damage may limit blood flow to a variety of organs and contribute to impaired organ function. The question is whether such impaired vascular function also occurs in brain.

### Cerebral micro-vessel structure in FASD

Our literature review identified two primary studies of brain microvessels, both of which examined postmortem fetal tissues from control cases and cases that met the diagnostic features of FAS or partial FAS (pFAS). The first study, which included 11 FAS/pFAS cases and eight control cases from gestational ages of 19–38 weeks (35), reported that the effects of PAE on brain vasculature were more prominent during later developmental periods. While control fetal brains exhibited a predominantly radial pattern of microvessels that traversed the marginal zone through the cortical plate and intermediate zones of the cerebral cortex, this radial pattern was significantly diminished in older fetuses with features of FAS/pFAS. The lack of observed effects in fetuses at earlier gestational ages suggests that vascular deficits may either result from cumulative PAE, or that the deficits from earlier episodes emerge later in development. More recently, the same research group published a second post-mortem study in four control fetuses and four fetuses with features of FAS/pFAS, ranging from gestational ages of 29–34 weeks (36). Using immunohistochemical staining for the microtubule-associated light chain protein, LC3 (Map1lc3a), the authors found a significant increase in LC3-positive puncta in endothelial cells lining cerebral microvasculature, an outcome that the authors interpreted as increased autophagy. Interestingly, in contrast to the previous study, increased LC3-positive puncta were observed early on, at gestational week 29, in fetuses with characteristics of FAS/pFAS, suggesting that autophagy may precede the loss of radial microvessels. A rigorous component of both studies was that the authors also replicated their findings in murine and cell culture models, suggesting that ethanol is a causal agent in the loss of brain microvessels, and that autophagy, a common stress response to nutrient deprivation, is one mediating factor.

An important caveat in interpreting these studies is their small sample sizes. Furthermore, fetuses affected by high levels of alcohol exposure (characterized as chronic daily alcohol exposure to binge levels) may have also resulted in spontaneous pregnancy termination. Other researchers have documented a link between heavy alcohol exposure during pregnancy and spontaneous pregnancy termination (37, 38). It is therefore possible that the heaviest alcohol exposures, which place pregnancy viability at risk, also compromise fetal vascular development. It remains to be determined if lower levels of prenatal alcohol exposure also compromise fetal brain vascular development in human populations. Another limitation of these studies is that they documented the acute effects of alcohol exposure in the fetus. For assessments in later life, a number of researchers turned to the retina, as a proxy tissue for assessing brain.

### Retinal circulation as a marker for cerebrovascular effects of FASD

Like brain, the retina is a central nervous system structure, protected by a structure equivalent to the blood-brain barrier, i.e., the blood-retinal barrier (39). As with the brain, the health of retinal neurons is dependent on an extensive microvascular network. A substantial advantage is that the structure and function of retinal arteries is readily accessible at any stage of postnatal life by standard ophthalmological visualization techniques. Not surprisingly, a number of studies on the vascular effects of PAE in human populations have focused on assessing retinal blood vessels. A majority of these studies, including some of the early case reports (40–42), reported increased tortuosity of retinal vessels (see Table 1).

**TABLE 1:** Summary of vascular studies in FASD in human populations.

FASD sample size (comparison group sample size)	Study type	Vascular region	Major findings	References
3 (0)	Case Study	Hepatic	Two cases of children with FAS presented with sclerosis of the central vein and distention, while 1 case displayed hepatic fibrosis and cystic kidney disease	(33)
2 (0)	Case Study	Heart and Lungs	Two infants with FAS presented with hypoplastic pulmonary arteries, congestive heart failure, and ventricular septal defects	(31)
17 (0)	Case Study	Eye	Children with FAS displayed retinal arterial and venous tortuosity, optic nerve hypoplasia, and decreased visual acuity	(40)
5 (0)	Case Study	Placental	There is significant endarteritis, trophoblastic basement membrane thickening, and basal lining hyperplasia in villous capillaries in placenta, in pregnancies that were prenatal alcohol-exposed	(34)
10 (0)	Case Study	Eye	Ten children with FAS or fetal alcohol effects (FAE) presented with ophthalmic defects including vessel tortuosity, optic nerve hypoplasia, cataracts, and visual impairment	(42)
16 (162)	Cohort Study	Eye	FASD associated with increased tortuosity of retinal vessels and decreased vessel branching	(43)
25 (92)	Cohort Study	Eye	117 children diagnosed with optic nerve hypoplasia were further assessed. 25 were also diagnosed with FAS, with some presenting with arterial and venous tortuosity	(44)
1 (0)	Case Study	Abdominal aorta	10-year-old boy with FAS and hypoplastic abdominal aorta presented with severe hypertension and was successfully treated with renal angioplasty	(32)
32 (25)	Cohort Study	Eye	In the sample, 30% showed retinal vessel tortuosity and 25% showed optic disc hypoplasia	(41)
31 (30)	Cohort Study	Brain	fMRI used to measure brain blood flow and blood oxygen levels. Persons with FASD exhibited significantly lower average activation for working memory tasks	(45)
4 (4)	Cohort Study	Brain	PAE increases the number of autophagic vacuoles in brain cortical microvessels	(36)
125 (500)	Cohort Study	Cardiovascular	Children with a diagnosis of FAS or partial FAS were at increased risk for hypertension compared to children in the general population (sampled from the National Health and Nutrition Examination Survey, NHANES)	(46)
37 (35)	Cohort Study	Eye	Observed association between prematurity and increased tortuosity of retinal vessels, strabismus, myopia, and optic nerve anomalies. Non-FASD cases were not further evaluated	(47)

In a larger study evaluating ophthalmologic findings in children with FASD, Gyllencreutz et al (47) assessed a cohort of 30–32 eastern European and Swedish children with FASD, who were longitudinally observed from childhood into adulthood for persisting ophthalmologic effects following PAE. The children had a median age of 7.9 years at the time that a multidisciplinary team diagnosed them with FASD, and evaluated visual acuity, stereoacuity, ocular media, strabismus, refraction, and fundus. At 13–18 years later (with the median age of study participants at 22 years old), the study cohort was reexamined and many of the earlier documented ophthalmologic findings - including astigmatism, defective stereoacuity, heterotropia, and optic nerve hypoplasia - were found to persist into adulthood. In addition, an increased tortuosity of retinal vessels was noted to persist into adulthood. Although none of the children enrolled in this study were born extremely premature, this study did include 12 children with a premature birth history (31–36 weeks of gestation), 16 children with a birth weight of less than 2,500 g, and 12 children who were born small for gestational age (SGA). These are important considerations, because, as we discuss later, prematurity is linked to increased tortuosity of retinal vessels, and other ophthalmological anomalies. A weakness of this study is that from the original cohort, children who did not receive a FASD diagnosis were not subsequently followed up. This means that it is difficult to ascertain whether the incidence of ophthalmological anomalies is higher in FASD populations compared to matched controls.

In contrast to the study presented above, another relatively large-scale study (48) compared 43 PAE children with 55 control children between the ages of 4 and 9, and found that both sampling groups exhibited an approximately equal incidence of arterial tortuosity (∼15–16%). This is an important and contrary finding, because it points to a limitation in case-report-type studies; specifically, that there was limited-to-no assessment of the frequency of vessel tortuosity in ‘control’ populations. However, this negative finding does not by itself disprove the linkage between PAE and vessel tortuosity. Control populations may well represent the heterogeneity of outcomes following prenatal experience, and a number of other factors that were not controlled for, including, for example, hypoxia, plasma hyperviscosity and hypercoagulability, [for review, see (49)], may result in an identical outcome. Importantly, prematurity, a condition with multiple etiologies including PAE, is also associated with retinal vessel tortuosity (50).

It is probable that ocular blood vessel pathology may not be a unique feature of FASD, as documented in an earlier 1999 study of Swedish children with different developmental complications. In that study by Hellstrom (43), children with a diagnosis of FAS (*n* = 16) were compared to those with other developmental complications, including preterm birth (*n* = 39), periventricular leukomalacia that is typically associated with perinatal hypoxia/ischemia (PVL, *n* = 17), and with septo-optic dysplasia with optic nerve hypoplasia and pituitary hormone insufficiency (*n* = 6). Children in these groups were compared to a cohort of “healthy white Swedish children” (*n* = 100). Digital image and fundoscopic analyses were used to examine optic nerve and retinal vessel morphology. In all groups, the study documented significantly increased tortuosity of retinal vessels (above the median for the reference cohort) and a lower number of branching points (below the median for the reference cohort). While this study showed that ocular vessel pathology was associated with a number of developmental pathologies, ∼43% of children with a diagnosis of FASD scored above the 95th percentile for the reference group. This outcome indicates that children with FASD are more likely to have retinal vessel pathology that the general population. This proportion was also larger than that for children with preterm birth. Contrary to the studies by Gyllencreutz et al. and Flanigan et al., discussed above, one of the strengths of this study was that FAS participants (median gestation age of 38 weeks at birth) and premature-birth participants (median gestational age of 29 weeks at birth) were separated for analysis. A weakness of this study was that the FAS sample in this study was poorly defined, except to refer to the then-current diagnostic guidelines as outlined by Sokol and Clarren (51). However, the Hellstrom study did support the specific linkage between a diagnosis of FAS and retinal vessel tortuosity, but also documented the linkage between other developmental anomalies and the same outcome.

In general, the findings from human studies support strong associations between prenatal alcohol exposure and vascular deficits, but cannot definitively advance a causal relationship between exposure and outcome. Studies of PAE in animal models, therefore, have a vital role in providing evidence for causality.

### Animal models of PAE

Early studies in animal models convincingly show that PAE is a causal factor in the cardiovascular defects that were described in human populations. For instance, in a 1986 study, Daft et al. (52) reported that two doses of ethanol on gestational day 8 in a pregnant mouse resulted in cardiac septal defects and defects in the cardiac outflow vessels, including the aorta. Moreover, these defects persisted and could be observed 10 days later, suggesting that the effects of an episode of PAE were permanent. A second publication from 2002 reported that PAE throughout gestation in a rat model, albeit at much lower levels than those reported in the previous study (∼24 mg/dL), resulted in a diminished vasoconstriction response of aortic rings following acute treatment with norepinephrine (53). The observed effects of PAE on the vasoconstrictive response were particularly strong when the arterial endothelium was intact, suggesting that the endothelium itself was a direct target of PAE. Interestingly, the study authors observed that PAE also resulted in diminished vasodilative response to the cholinergic mimetic carbamylcholine chloride, suggesting a broader impact of PAE on the adaptability of large arteries to physiological demand. Collectively, these data identify PAE, over a range of doses and exposure times, as a causal factor in the development of persistent structural and functional cardiovascular defects.

Other studies in primate (54), ovine (55), and rodent models (56) also point to placental and uterine blood flow as targets of prenatal ethanol, relating vascular deficiencies in these tissues to decreased fetal growth (see Table 2). However, cardiac defects due to developmental ethanol exposure have also been documented in non-placental vertebrate models, like zebrafish (66), suggesting that ethanol’s effects on cardiovascular development are not exclusively mediated by potential utero-placental insufficiency. Again, these studies support a causal link between PAE, decreased peripheral blood flow, and subsequent deficits and brain growth. However, the first studies that specifically investigate PAE effects on brain vasculature were not published until approximately 30 years after FAS was first described in human populations (7) and ∼25 years after it was first described in the United States (6). This delay in research modeling alcohol’s effects on brain vasculature speaks to the neural cell-centric focus of the research field at that stage.

**TABLE 2:** Studies on the vascular effects of PAE in animal models.

Species	Study type	Vascular region	Major findings	References
Sheep	*In vivo* and *in vitro*	Brain	PAE in early gestation resulted in decreased postnatal cerebral blood flow in labs, in response to both hypo- and hypercapnia, compared to controls	(57)
Rat	*In vivo* and *in vitro*	Aorta	PAE alters aortic vascular contractile function, decreased vasoconstrictive response to norepinephrine and potassium chloride	(53)
Sheep	*In vivo*	Brain	In the presence of acidemia and hypercapnia, without hypoxia, there was increased blood flow to brain in sheep that were exposed to moderate alcohol levels during late gestation	(58)
Sheep	*In vivo*	Brain	PAE in mid gestation significantly attenuated dilatory cerebral blood flow response to hypoxia	(59)
Sheep	*In vivo*	Brain	PAE resulted in increased maximum vasodilation of fetal cerebral arterioles and vessel response to selective A2A adenosine receptor agonist and to acidosis	(60)
Sheep	*In vivo*	Brain	PAE in mid-gestation increased the dilatory response of the adult intracerebral arteries due to VIP but had no difference in response to pH or myogenic tone	(61)
Sheep	*In vivo*	Brain	PAE in the mid-gestation resulted in lower brain weight, but no significant differences in cerebral microvessel density	(62)
Sheep	*In vitro*	Uterine	Isolated uterine endothelial cells from pregnant ewes, exposed to binge-like ethanol levels. Observed decreased eNOS expression, phosphorylation and expression of eNOS related proteins	(55)
Mice	*In vivo*	Placental, cardiac	Binge-like PAE in pregnant mice at gastrulation persistently increases vascular resistance in umbilical artery, cardiac valvular regurgitation and isovolemic relaxation time	(63)
Mice	*In vivo*	Brain	Single and repeated binge-like PAE in mid- to late-pregnancy, during the peak period of cortical neurogenesis results in persistent decrease in cardiac output through umbilical and cerebral arteries	(64)
Mice	*In vivo* and *ex vivo*	Brain	PAE in the late gestation resulted in disorganized cerebral microvascular networks, including reduced density of cortical vasculature, decreased VEGF and VEGF receptor mRNA and increased VEGF receptor (R1) expression and *ex vivo*, decreased in microvessel plasticity	(35)
Rat	*In vivo*	Uterine	An episode of PAE in both early and late pregnancy decreased acetylcholine-induced uterine artery vasodilation	(65)
Zebrafish	*In vivo*	Cardiac	Exposure during embryogenesis resulted in a dose-related increase in irreversible damage to dorsal aorta, segmental artery coarctation, and motor function deficits	(66)
Primate	*In vivo*	Placental	PAE in early pregnancy in Rhesus monkeys significantly decreased placental perfusion and oxygenation in fetal vasculature in later stages of pregnancy	(54)
Primate	*In vivo* and *ex vivo*	Brain	Exposure of fetal MCAs to alcohol in mid pregnancy induces increased dilation of cerebral arteries and peak systolic velocity, mediated by vascular endocannabinoid receptors	(67)
Mouse	*In vivo*	Carotid artery	The carotid arteries of adult mice with PAE exhibited significantly decreased blood acceleration with loss of blood flow to the brain in the long term, and was associated with decreased recovery from cerebrovascular stroke	(68)
Mouse and Human	*In vivo*	Placenta, Brain	Deficiency in placental angiogenic factor implicated in VEGF (vascular endothelial growth factor)-receptor mediated deficiencies in brain angiogenesis	(69)
Primate	*In vivo* and *ex vivo*	Eye	PAE resulted in increased intraocular pressure (IOP) in juvenile and adult offspring, increased fundal tessellation indicative of abnormal choroidal vascularization, and astrocytosis	(70)
Primate	*In vivo*	Brain	Peak systolic velocity and pulsatility index of anterior and middle cerebral arteries decreased during episodes of alcohol intoxication, with decreased fetal cerebral artery Doppler indices	(71)
Rats	*In vivo*	Uterine	Uterine arteries from alcohol exposed rats had reduced acetylcholine-dependent relaxation and impaired endothelial nitric oxide signaling	(72)
Mouse	*In vivo*	Brain	*In utero* speckle variance optical coherence angiography showed that binge-like PAE caused rapid and significant cerebral microvessel constriction compared to controls	(73)
Primate	*In vivo*	Brain	PAE in mid pregnancy resulted in significant increases in transferase and oxidoreductase class proteins and increased ALDH activity in fetal cerebral basilar arteries	(74)
Primate	*In vivo* and *ex vivo*	Brain	PAE induced fetal artery dilation mediated by cannabinoid signaling is transient and does not persist to the end of pregnancy	(75)
Rat	*In vivo*	Brain	PAE throughout pregnancy resulted in decreased nitric oxide dependent dilation of cerebral arterioles, higher superoxide levels, increased brain infarct volume following cerebrovascular ischemia, and increased levels of superoxide	(76)
Rat	*In vivo*	Brain	PAE throughout gestation resulted in decreased stimulus-dependent vasodilation response in cerebral arteries in young adult offspring	(76)
Mouse	*In vivo*	Brain	*In utero* ethanol-exposure resulted in an acute-onset dose-dependent decrease in cerebral microvessel diameter and decreased blood flow measured by correlation mapping optical coherence angiography. Concurrently the maternal femoral artery exhibited vasodilation	(77)
Mouse	*In vivo*	Umbilical	PAE in the latter half of gestation resulted in intrauterine growth retardation, and diminished umbilical arterial blood flow, assessed by pulse-wave Doppler ultrasound. RNA-seq analysis of the placenta, this study found diminished expression of placental genes for hematopoiesis and chemosensory pathways	(56)

### Early assessments of cerebral circulation in animal models of PAE

In 1997, Gleason et al. at Johns Hopkins University, School of Medicine used a sheep model of PAE (57), in which pregnant ewes received either an alcohol or saline infusion daily for 3 weeks during early gestation (∼Gestational Day 31). This exposure resulted in mean blood ethanol concentrations of ∼167 mg/dL after 1 hour. Physiological characteristics of cerebral blood were ascertained by sampling from the superior sagittal sinus of instrumented PAE and control newborn lambs, and blood flow was quantified using infusions of radionuclide-labeled microspheres. The authors reported that both the hypo- and hypercapnic cerebral blood flow response was decreased in PAE lambs compared to controls.

Subsequently, Parnell et al. at Texas A&M University (58) and Mayock et al. at the University of Washington (59) published the next preclinical studies on the effects of PAE on blood flow in the fetal brain, also using an ovine model. Notably, in both studies, assessments of PAE effects were conducted in the fetus, i.e., more proximate to the exposure period, rather than postnatally, as reported above by Gleason et al. In the study by Parnell et al., pregnant ewes were exposed to ethanol between gestational days (GD) 109–132, resulting in maternal blood ethanol concentrations between 85–185 mg/dL. The authors also used radionuclide-labeled microspheres to assess cerebral blood flow, and found increased retention of radionuclides in the fetal cerebellum, indicative of increased blood flow, but only at the highest levels of exposure.

Studies by Mayock et al. at the University of Washington, Seattle, also used an ovine model to assess the effects of PAE on brain vasculature (59, 60). In their studies, Mayock et al. exposed pregnant ewes to alcohol at an earlier time frame than the previously cited paper by Parnell et al. PAE occurred *via* daily intravenous infusion between GD 60–90, equivalent to the 2nd trimester period in human pregnancy. Post-infusion maternal alcohol levels peaked at ∼200–214 mg/dL, which although high, are within the range of levels attained by individuals with alcohol use disorders [e.g., see (78)]. In their 2007 study, Mayock et al. utilized a similar radioisotope retention paradigm to assess cerebral blood flow as used by Parnell et al. However, this study found decreased cerebral blood flow due to PAE, opposite of what was previously reported.

In their subsequent 2008 report (60), Mayock et al. isolated penetrating fetal cerebral arterioles arising from the pial surface of fetal lambs between GD 125–128, investigating the persistent effects of on PAE offspring compared to control, saline infusion-exposed fetuses. The authors reported two key findings: more than 5 weeks after the final exposure, cerebral arterioles from PAE fetuses exhibited 1) a significant dilatory response to decreased pH, and 2) an increased maximal dilatory response to an Adenosine A2 agonist, CGS-21680. A follow-up study by the same team (61), also in isolated fetal sheep cerebral arteries, reported on a similar PAE-induced increase in vasodilatory response, this time to vasoactive intestinal peptide, suggesting that PAE may result in a general enhancement of stimulus-dependent cerebral arterial vasodilation.

A more recent study in a non-human primate model of PAE used an *ex vivo* model of pressurized fetal cerebral arteries (67), demonstrating that acute ethanol exposure also results in rapid vessel dilation. In this primate study, the authors were not able to document additional effect of prior intragastric PAE directly on the dilation response, though they did document increased sensitivity to cannabinoid signaling due to PAE. However, this last study also used a more limited exposure—just three episodes of intragastric gavage during the 2nd trimester-equivalent period of human pregnancy—and the peak maternal blood alcohol levels attained were ∼80 mg/dL, substantially lower than levels attained in the first two studies.

Collectively, the aforementioned studies demonstrate that PAE can influence cerebral blood flow regardless of whether exposure occurred in early, mid- or late pregnancy. Moreover, vascular effects can persist beyond the period of ethanol exposure, into the neonatal period. Importantly, they also showed that vasodilation, via ethanol or other dilatory stimuli, was a long term response to PAE in cerebral blood vessels. However, the outcome of PAE for brain circulation itself is still unclear, since the radionuclide retention studies by Parnell et al. (58) were interpreted by the authors to suggest increased flow (at least in the posterior, cerebellar circulation), whereas Mayock et al. (59) and earlier, Gleason et al. (57), interpreted their data to indicate decreased blood flow, and under conditions of increased demand, reduced oxygen delivery within the brain. Some of the differences in study outcomes reported above, may well be due to inter-study differences in the developmental timing of alcohol exposure and outcome assessment, as well as brain regional differences. It should also be noted that, radionuclide-labeled microsphere retention methodology which was historically, the gold standard for assessing end-organ vascular perfusion, was nevertheless subject to interpretive limitations. Based on the empirical association between afferent arterial tone and end-organ perfusion, increased brain retention of radio-label is causally linked to peripheral arterial vasodilation (57, 58). However, this interpretation requires some caution, since it does not account for potential dilation of efferent vessels, microcirculatory arterial-venous anastomotic shunts which are present in brain parenchyma [e.g., see (79)], that may result in increased clearance and decreased tissue retention of radiolabel (80), accounting for some discrepancies in study outcomes outlined above. Recent studies have used more direct, *in vivo* imaging modalities such as ultrasound imaging and optical coherence tomography to assess brain blood flow in response to PAE.

### Ultrasound studies of major cerebral arteries in animal models

Studies using ultrasound imaging in primate (67, 71) and rodent models (64, 68) have been successful in visualizing flow parameters in large cerebral arteries like the anterior (ACA), middle (MCA) and posterior (PCA) cerebral arteries. Both primate and rodent models indicate that the immediate and persistent effect of PAE is decreased cerebral blood flow as measured by peak systolic velocity, or velocity time integral [VTI, a composite index that is a measure of the cardiac output through the assessed cerebral vessel (81)]. However, studies in primate models generally indicate that the effects of PAE do not persist past the exposure period, and specifically, not to pregnancy term. For example, Tobiasz et al. (71) found that fetal baboons exposed to PAE experienced decreased systolic velocity in anterior and middle cerebral arteries during the acute period of intoxication, but that these vascular effects did not persist through gestation. However, in rodent (mouse) models, Bake et al. showed that the PAE effect of decreased velocity, and decreased VTI could persist through gestation (64). In contrast to the acute fetal effects of PAE, Bake et al., in a follow-up study (68), showed that young adult PAE offspring (3 months of age) exhibited significantly increased carotid artery VTI, indicative of a vasoconstrictive response. It is certainly possible that species differences may have contributed to differences in the persistence of the vascular effects of PAE, though the observation of a peripheral hypertensive phenotype in human populations of children and teenagers with FASD (46), is consistent with increased carotid VTI in young adult PAE offspring in the above mouse study, and argues against a role for species differences. A second and plausible explanation is that the persistence of PAE effects had more to do with the dose and frequency of ethanol exposure. Blood alcohol levels of ∼80 mg/dL were attained in the primate studies, for example, with exposures spaced 10 days apart (71), whereas BACs of 117–150 mg/dL were reached in the mouse, with up to two exposures per day for a 4-day exposure window (68). It is possible therefore that the cumulative exposure in the mouse model was significantly higher than that attained in the primate model. Nevertheless, both models show that, at least immediately, there is a net decline in fetal cerebral circulation due to PAE, consistent with the hypothesis that, in the short term, alcohol induces vasodilation (71). It is important to note that Bake et al. also followed PAE and control offspring into mature adulthood (12 months of age), where PAE had a diametrically opposite effect compared to that observed in young adults, i.e., resulting in decreased VTI compared to control offspring (68). This outcome suggests that in the long term, PAE may result in cranial vascular hypoperfusion in middle-aged adults, though at this time, there is no comparable data on vascular health in middle-aged persons with FASD.

### Analysis of cerebral microcirculation in animal models

More recently, optical coherence angiography has been used to document the effects of PAE on blood flow through even smaller cerebral arteries and arterioles, including those that serve as tributaries from the ACA and MCA (73, 77). These studies report that PAE results in a rapid and dose-dependent decrease in the diameter of fetal cerebral micro-vessels, whereas the maternal femoral artery experienced vasodilation in the same time frame, as expected. It is likely that the vasoconstrictive response of cerebral microvessels is a compensatory adaptation to the dilation of larger and afferent vessels, as a means to maintain pressure in the microvascular network. This compensatory effort may be at least partly successful, as suggested by the increased radionuclide retention in the cerebellum that observed by Parnell et al. previously [as outlined above (58)].

Anatomical studies in mouse models also provide supporting evidence that PAE results in persistent damage to the intracerebral microvasculature. For instance, Jegou et al. (35) used a PAE model, albeit with heavy ethanol exposure, daily through the second half of murine gestation, and observed that mice with PAE had reduced density of cortical vasculature and disordered micro-vessel orientation, which were normally radially-oriented in control mice progeny. These data cumulatively suggest that even if the intracerebral microvasculature adapt and compensate for PAE, the immediate and long-term outcomes are likely to be vascular insufficiency within the brain. It will be important to ascertain whether this insufficiency extends to the availability of collateral circulation between terminal branches of cerebral arteries like the MCA, ACA and PCA. The evidence suggests that collateral circulation may also be impaired, and that vascular insufficiency may also result in long-term adverse consequences to adult-onset brain diseases. For instance, two studies have documented the PAE results in decreased neurological recovery from an episode of cerebrovascular ischemic stroke (68, 76). While the study by Bake et al. (68) did not document companion damage to brain tissue despite neurological deficits, the study by Canzi and Mayhan (76) showed that ischemia did result in increased stroke volume in PAE offspring. Therefore, it is likely that compensatory collateral circulation is also impaired following PAE. Importantly, collateral circulation can emerge rapidly, during the period of cerebral arterial occlusion itself (82), suggesting the presence of latent collateral tributaries within leptomeningeal tissues. It is likely, though there is a need for further investigation, that latent collateral circulation is also impaired in PAE, leading to worse stroke outcomes.

### Retinal imaging in animal models

As with human studies, recent studies in animal (primate) models, have also focused an imaging retinal vasculature to assess the effects of PAE on brain blood flow in adult offspring. One study in a large cohort of vervet monkeys (29 PAE and 20 controls), maintained as a community (70), investigated the effect of exposure of ethanol *in utero* on retinal abnormalities and premature aging of the retina using *in vivo* examination of the fundus and intraocular pressure (IOP), as well as a number of physiologic and anatomic measures. This study had well-defined and extensive data on maternal alcohol consumption for each pregnancy, with a range of exposure from 1.2 to 5.51 g of alcohol/kg body weight/day from mid-pregnancy to term. The authors found that PAE resulted in increased IOP, increased fundal vascularization, and the retina showed increased evidence for astrocytosis, particularly in the retinal ganglion cell layer and optic nerve, suggesting retinal damage. This is potentially an important finding, since increased IOP may be indicative of ocular hypertension, a condition linked to decreased ocular blood flow and thinning of the choroid plexus in human populations (83), and to increased risk for glaucoma (84). Collectively, the studies cited earlier in human populations, as well as the above study by Bouskila et al., make a case for monitoring ocular health in FASD populations, not only to decrease the risk for diseases like glaucoma, but potentially as a biomarker for neurovascular health.

### Mechanisms that mediate effects of PAE on the vascular system

Several studies, including many cited above, have taken the first important steps to uncover mechanisms of ethanol toxicity. One consensus finding is that PAE alters the vasodilation response to G-protein receptor coupled signaling mechanisms and nitric oxide signaling [e.g., (53, 59, 60, 67, 72, 85)], as well as other environmental stressors (76). Furthermore, studies do support the theory that PAE also interferes with the trajectory and development of brain microvessels. This implicates the potential role of angiogenic factors, such as signaling through the vascular endothelial growth factor (VEGF) system. Lecuyer et al. (69) found evidence for decreased VEGF signaling and decreased brain angiogenesis due to diminished levels of placental-derived VEGF family member, PLGF (Placenta Growth Factor). Interestingly, PLGF supplementation was able to ameliorate the effects of PAE in their mouse model. Autophagy has also been implicated as a molecular mechanism in the potential remodeling of cerebro-vasculature following PAE (36) in both human studies and mouse models. Autophagy is an important stress response to vascular injury (reviewed in (86), and a potential target for therapeutic intervention. However, much more research is needed to identify and track mechanistic linkages between PAE and brain vascular outcomes.

## Conclusion and limitations

Although the research on PAE effects on developing vasculature, and in particular brain vasculature, are somewhat sparse, both human studies and studies in animal models document the deleterious and persistent effects of PAE on cerebrovascular structure and function. The current research studies are based on relatively small sample sizes, but collectively provide a preponderance of evidence pointing towards several important pathways related to vasodilation, oxidative stress, and flow dynamics. It is also clear that vascular deficits are likely to persist through the lifetime and contribute to risks for adverse outcomes following adult-onset disease, like cerebrovascular stroke. In this context, one study which documented significantly increased risk for hypertension in children and adolescents with a diagnosis of FAS or pFAS (46) is particularly important, since it suggests that the risk for adult-like cardiovascular disease may appear earlier in persons with FASD compared to the general population, and further implicates pituitary and renal dysfunction in the pathogenesis of FASD. For instance, studies in human populations have linked prenatal alcohol exposure to hypermethylation of the proopiomelanocortin (POMC) locus (87), and in animal studies, PAE has been shown to result in deficits in feed-back inhibition and hyperresponsivity of the hypothalamic-pituitary-adrenal (HPA) axis in affected offspring [e.g., see (88)]. Animal studies have also shown that PAE results in loss of renal nephrons and increased blood pressure in exposed offspring (89). Collectively these data suggest that hyperresponsiveness of the HPA axis and renal deficiency may contribute to vascular pathology in FASD, and support the need for further studies on the intersection between endocrine, renal and vascular function.

Human studies document disruption to mammalian target of rapamycin (mTOR) pathways, with increased autophagic vacuoles in brain microvessels, deficits in mitochondrial pathways, and basement membrane adaptations, including capillary basal hyperplasia and endarteritis. All of these outcomes are compatible with a stress-related remodeling of tissue microvasculature following PAE that likely results in compromised vascular function. Animal models further provided evidence that PAE is a causal factor in vascular deficits, including those of the cerebral vasculature. Additionally, preclinical research has identified PAE-affected mechanisms of signaling pathways that target vasodilation, such as the endocannabinoid receptor system, prostacyclin and nitric oxide pathways, and calcium mobilization. Other factors, such as impaired VEGF receptor signaling and changes in protein expression related to mitochondria and oxidative stress, may contribute further to structural pathologies.

Our analysis of the literature also identified studies in both human and animal populations that assessed ocular vascular function following PAE. Studies in FASD populations documented other ophthalmological findings, such as increased retinal vessel tortuosity, which should be readily assessable in primary healthcare settings. These studies make a case that routine, easy to accomplish, clinical assessments of ocular blood flow and vascular structure may be useful as a proxy marker for neurovascular competency in persons with diagnoses along the FASD continuum. Additional studies are needed to determine whether such routine ocular assessments have predictive value for the management of adult-onset cerebrovascular disease in FASD populations. However, attention to cardiovascular and cerebrovascular health in FASD populations is likely to be helpful in managing both early cognitive and neurobehavioral deficits as well the delayed, adult consequences of PAE.

## Data Availability

The original contributions presented in the study are included in the article/supplementary material, further inquiries can be directed to the corresponding author.

## References

[1] Astley SJ Olson HC Kerns K Brooks A Aylward EH Coggins TE Neuropyschological and behavioral outcomes from a comprehensive magnetic resonance study of children with fetal alcohol spectrum disorders. Can J Clin Pharmacol (2009) 16(1):e178–201. 19329824 PMC4188550

[2] Hoyme HE Kalberg WO Elliott AJ Blankenship J Buckley D Marais AS Updated clinical guidelines for diagnosing fetal alcohol spectrum disorders. Pediatrics (2016) 138(2):e20154256. 10.1542/peds.2015-4256 27464676 PMC4960726

[3] Carter RC Jacobson JL Molteno CD Dodge NC Meintjes EM Jacobson SW. Fetal alcohol growth restriction and cognitive impairment. Pediatrics (2016) 138(2):e20160775. 10.1542/peds.2016-0775 27401098 PMC4960732

[4] Clarren SK Alvord EC Jr. Sumi SM Streissguth AP Smith DW. Brain malformations related to prenatal exposure to ethanol. J Pediatr (1978) 92(1):64–7. 10.1016/s0022-3476(78)80072-9 619080

[5] Hanson JW Jones KL Smith DW. Fetal alcohol syndrome. Experience with 41 patients. Jama (1976) 235(14):1458–60. 10.1001/jama.235.14.1458 946444

[6] Jones KL Smith DW Ulleland CN Streissguth P. Pattern of malformation in offspring of chronic alcoholic mothers. Lancet (1973) 1(7815):1267–71. 10.1016/s0140-6736(73)91291-9 4126070

[7] Lemoine P Harouseau H Borteryu JT Menuet JC. Les enfants des parents alcooliques: Anomalies observees apropos de 127 cas. Ouest Med (1968) 21:476–82.

[8] Norman AL Crocker N Mattson SN Riley EP. Neuroimaging and fetal alcohol spectrum disorders. Dev Disabil Res Rev (2009) 15(3):209–17. 10.1002/ddrr.72 19731391 PMC3442778

[9] Fryer SL McGee CL Matt GE Riley EP Mattson SN. Evaluation of psychopathological conditions in children with heavy prenatal alcohol exposure. Pediatrics (2007) 119(3):e733–41. 10.1542/peds.2006-1606 17332190

[10] Schonfeld AM Mattson SN Lang AR Delis DC Riley EP. Verbal and nonverbal fluency in children with heavy prenatal alcohol exposure. J Stud Alcohol (2001) 62(2):239–46. 10.15288/jsa.2001.62.239 11327190

[11] Vaurio L Riley EP Mattson SN. Differences in executive functioning in children with heavy prenatal alcohol exposure or attention-deficit/hyperactivity disorder. J Int Neuropsychol Soc (2008) 14(1):119–29. 10.1017/S1355617708080144 18078538 PMC3713496

[12] Hagan JF Jr. Balachova T Bertrand J Chasnoff I Dang E Fernandez-Baca D Neurobehavioral disorder associated with prenatal alcohol exposure. Pediatrics (2016) 138(4):e20151553. 10.1542/peds.2015-1553 27677572 PMC5477054

[13] Kable JA O'Connor MJ Olson HC Paley B Mattson SN Anderson SM Neurobehavioral disorder associated with prenatal alcohol exposure (ND-PAE): Proposed DSM-5 diagnosis. Child Psychiatry Hum Dev (2016) 47(2):335–46. 10.1007/s10578-015-0566-7 26202432

[14] Bakhireva LN Sharkis J Shrestha S Miranda-Sohrabji TJ Williams S Miranda RC. Prevalence of prenatal alcohol exposure in the state of Texas as assessed by phosphatidylethanol in newborn dried blood spot specimens. Alcohol Clin Exp Res (2017) 41(5):1004–11. 10.1111/acer.13375 28294365

[15] Umer A Lilly C Hamilton C Baldwin A Breyel J Tolliver A Prevalence of alcohol use in late pregnancy. Pediatr Res (2020) 88(2):312–9. 10.1038/s41390-019-0731-y 31899916 PMC7384987

[16] DiBattista A Ogrel S MacKenzie AE Chakraborty P. Quantitation of phosphatidylethanols in dried blood spots to determine rates of prenatal alcohol exposure in Ontario. Alcohol Clin Exp Res (2022) 46(2):243–51. 10.1111/acer.14766 34939205

[17] Baldwin AE Hayes N Ostrander E Magri R Sass N Dos Anjos Mesquita M Phosphatidylethanol levels in postpartum women and their newborns in Uruguay and Brazil. Alcohol Clin Exp Res (2020) 44(6):1292–9. 10.1111/acer.14339 32441809 PMC7310578

[18] May PA Chambers CD Kalberg WO Zellner J Feldman H Buckley D Prevalence of fetal alcohol spectrum disorders in 4 US communities. Jama (2018) 319(5):474–82. 10.1001/jama.2017.21896 29411031 PMC5839298

[19] Roozen S Peters GJ Kok G Townend D Nijhuis J Curfs L. Worldwide prevalence of fetal alcohol spectrum disorders: A systematic literature review including meta-analysis. Alcohol Clin Exp Res (2016) 40(1):18–32. 10.1111/acer.12939 26727519

[20] Norman MG O'Kusky JR. The growth and development of microvasculature in human cerebral cortex. J Neuropathol Exp Neurol (1986) 45(3):222–32. 10.1097/00005072-198605000-00003 3958756

[21] Madelaine R Sloan SA Huber N Notwell JH Leung LC Skariah G MicroRNA-9 couples brain neurogenesis and angiogenesis. Cell Rep (2017) 20(7):1533–42. 10.1016/j.celrep.2017.07.051 28813666 PMC5665055

[22] Sathyan P Golden HB Miranda RC. Competing interactions between micro-RNAs determine neural progenitor survival and proliferation after ethanol exposure: Evidence from an *ex vivo* model of the fetal cerebral cortical neuroepithelium. J Neurosci (2007) 27(32):8546–57. 10.1523/JNEUROSCI.1269-07.2007 17687032 PMC2915840

[23] Pappalardo-Carter DL Balaraman S Sathyan P Carter ES Chen WJ Miranda RC. Suppression and epigenetic regulation of MiR-9 contributes to ethanol teratology: Evidence from zebrafish and murine fetal neural stem cell models. Alcohol Clin Exp Res (2013) 37(10):1657–67. 10.1111/acer.12139 23800254 PMC3785568

[24] Chen KC Song ZM Croaker GD. Brain size reductions associated with endothelin B receptor mutation, a cause of Hirschsprung's disease. BMC Neurosci (2021) 22(1):42. 10.1186/s12868-021-00646-z 34147087 PMC8214790

[25] Fan J Taylor PA Jacobson SW Molteno CD Gohel S Biswal BB Localized reductions in resting-state functional connectivity in children with prenatal alcohol exposure. Hum Brain Mapp (2017) 38(10):5217–33. 10.1002/hbm.23726 28734059 PMC6377933

[26] Fryer SL Tapert SF Mattson SN Paulus MP Spadoni AD Riley EP. Prenatal alcohol exposure affects frontal-striatal BOLD response during inhibitory control. Alcohol Clin Exp Res (2007) 31(8):1415–24. 10.1111/j.1530-0277.2007.00443.x 17559542

[27] Lindinger NM Jacobson JL Warton CMR Malcolm-Smith S Molteno CD Dodge NC Fetal alcohol exposure alters BOLD activation patterns in brain regions mediating the interpretation of facial affect. Alcohol Clin Exp Res (2021) 45(1):140–52. 10.1111/acer.14519 33220071 PMC7970581

[28] Mark CI Mazerolle EL Chen JJ. Metabolic and vascular origins of the BOLD effect: Implications for imaging pathology and resting-state brain function. J Magn Reson Imaging (2015) 42(2):231–46. 10.1002/jmri.24786 25727523

[29] Jones KL Smith DW. The fetal alcohol syndrome. Teratology (1975) 12(1):1–10. 10.1002/tera.1420120102 1162620

[30] Barth H Schmaltz AA Steil E Apitz J. Combination of ventricular septal defect with unilateral pulmonary artery stenoses and contralateral pulmonary hypertension. Z Kardiol (1984) 73(11):710–6. 6543069

[31] Steeg CN Woolf P. Cardiovascular malformations in the fetal alcohol syndrome. Am Heart J (1979) 98(5):635–7. 10.1016/0002-8703(79)90290-4 158960

[32] Cura MA Bugnone A Becker GJ. Midaortic syndrome associated with fetal alcohol syndrome. J Vasc Interv Radiol (2002) 13(11):1167–70. 10.1016/s1051-0443(07)61960-5 12427818

[33] Habbick BF Zaleski WA Casey R Murphy F. Liver abnormalities in three patients with fetal alcohol syndrome. Lancet (1979) 1(8116):580–1. 10.1016/s0140-6736(79)91007-9 85166

[34] Amankwah KS Kaufmann RC. Ultrastructure of human placenta: Effects of maternal drinking. Gynecol Obstet Invest (1984) 18(6):311–6. 10.1159/000299099 6519561

[35] Jegou S El Ghazi F de Lendeu PK Marret S Laudenbach V Uguen A Prenatal alcohol exposure affects vasculature development in the neonatal brain. Ann Neurol (2012) 72(6):952–60. 10.1002/ana.23699 23280843

[36] Girault V Gilard V Marguet F Lesueur C Hauchecorne M Ramdani Y Prenatal alcohol exposure impairs autophagy in neonatal brain cortical microvessels. Cell Death Dis (2017) 8(2):e2610. 10.1038/cddis.2017.29 28182007 PMC5386476

[37] Bailey BA Sokol RJ. Prenatal alcohol exposure and miscarriage, stillbirth, preterm delivery, and sudden infant death syndrome. Alcohol Res Health (2011) 34(1):86–91. 23580045 PMC3860553

[38] Chiodo LM Bailey BA Sokol RJ Janisse J Delaney-Black V Hannigan JH. Recognized spontaneous abortion in mid-pregnancy and patterns of pregnancy alcohol use. Alcohol (2012) 46(3):261–7. 10.1016/j.alcohol.2011.11.006 22440690 PMC3354912

[39] Cunha-Vaz J Bernardes R Lobo C. Blood-retinal barrier. Eur J Ophthalmol (2011) 21(6):S3–9. 10.5301/EJO.2010.6049 23264323

[40] Gonzalez ER. New ophthalmic findings in fetal alcohol syndrome. Jama (1981) 245(2):108. 10.1001/jama.1981.03310270004002 7452823

[41] Ribeiro IM Vale PJ Tenedorio PA Rodrigues PA Bilhoto MA Pereira HC. Ocular manifestations in fetal alcohol syndrome. Eur J Ophthalmol (2007) 17(1):104–9. 10.1177/112067210701700114 17294389

[42] Stromland K Sundelin K. Paediatric and ophthalmologic observations in offspring of alcohol abusing mothers. Acta Paediatr (1996) 85(12):1463–8. 10.1111/j.1651-2227.1996.tb13953.x 9001659

[43] Hellstrom A. Optic nerve morphology may reveal adverse events during prenatal and perinatal life-digital image analysis. Surv Ophthalmol (1999) 44(1):S63–73. 10.1016/s0039-6257(99)00067-3 10548118

[44] Hellstrom A Wiklund LM Svensson E. The clinical and morphologic spectrum of optic nerve hypoplasia. J AAPOS (1999) 3(4):212–20. 10.1016/s1091-8531(99)70005-4 10477223

[45] Gautam P Nunez SC Narr KL Mattson SN May PA Adnams CM Developmental trajectories for visuo-spatial attention are altered by prenatal alcohol exposure: A longitudinal fmri study. Cereb Cortex (2015) 25(12):4761–71. 10.1093/cercor/bhu162 25092900 PMC4635917

[46] Cook JC Lynch ME Coles CD. Association analysis: Fetal alcohol spectrum disorder and hypertension status in children and adolescents. Alcohol Clin Exp Res (2019) 43(8):1727–33. 10.1111/acer.14121 31166027

[47] Gyllencreutz E Aring E Landgren V Svensson L Landgren M Gronlund MA. Ophthalmologic findings in fetal alcohol spectrum disorders - a cohort study from childhood to adulthood. Am J Ophthalmol (2020) 214:14–20. 10.1016/j.ajo.2019.12.016 31926885

[48] Flanigan EY Aros S Bueno MF Conley M Troendle JF Cassorla F Eye malformations in children with heavy alcohol exposure *in utero* . J Pediatr (2008) 153(3):391–5. 10.1016/j.jpeds.2008.04.024 18571671 PMC2570183

[49] Vilela MA Amaral CE Ferreira MAT. Retinal vascular tortuosity: Mechanisms and measurements. Eur J Ophthalmol (2021) 31(3):1497–506. 10.1177/1120672120979907 33307777

[50] International Committee for the Classification of Retinopathy of P. The international classification of retinopathy of prematurity revisited. Arch Ophthalmol (2005) 123(7):991–9. 10.1001/archopht.123.7.991 16009843

[51] Sokol RJ Clarren SK. Guidelines for use of terminology describing the impact of prenatal alcohol on the offspring. Alcohol Clin Exp Res (1989) 13(4):597–8. 10.1111/j.1530-0277.1989.tb00384.x 2679217

[52] Daft PA Johnston MC Sulik KK. Abnormal heart and great vessel development following acute ethanol exposure in mice. Teratology (1986) 33(1):93–104. 10.1002/tera.1420330112 3738814

[53] Turcotte LA Aberle NS Norby FL Wang GJ Ren J. Influence of prenatal ethanol exposure on vascular contractile response in rat thoracic aorta. Alcohol (2002) 26(2):75–81. 10.1016/s0741-8329(01)00198-7 12007582

[54] Lo JO Schabel MC Roberts VH Wang X Lewandowski KS Grant KA First trimester alcohol exposure alters placental perfusion and fetal oxygen availability affecting fetal growth and development in a non-human primate model. Am J Obstet Gynecol (2017) 216(3):302 e1–302302.e8. 10.1016/j.ajog.2017.01.016 PMC5334435 28153658

[55] Ramadoss J Jobe SO Magness RR. Alcohol and maternal uterine vascular adaptations during pregnancy-part I: Effects of chronic *in vitro* binge-like alcohol on uterine endothelial nitric oxide system and function. Alcohol Clin Exp Res (2011) 35(9):1686–93. 10.1111/j.1530-0277.2011.01515.x 21599719 PMC4241777

[56] Pinson MR Tseng AM Adams A Lehman TE Chung K Gutierrez J Prenatal alcohol exposure contributes to negative pregnancy outcomes by altering fetal vascular dynamics and the placental transcriptome. Alcohol Clin Exp Res (2022) 46(6):1036–49. 10.1111/acer.14846 35474222 PMC9325399

[57] Gleason CA Iida H Hotchkiss KJ Northington FJ Traystman RJ. Newborn cerebrovascular responses after first trimester moderate maternal ethanol exposure in sheep. Pediatr Res (1997) 42(1):39–45. 10.1203/00006450-199707000-00007 9212035

[58] Parnell SE Ramadoss J Delp MD Ramsey MW Chen WJ West JR Chronic ethanol increases fetal cerebral blood flow specific to the ethanol-sensitive cerebellum under normoxaemic, hypercapnic and acidaemic conditions: Ovine model. Exp Physiol (2007) 92(5):933–43. 10.1113/expphysiol.2007.038091 17526556

[59] Mayock DE Ness D Mondares RL Gleason CA. Binge alcohol exposure in the second trimester attenuates fetal cerebral blood flow response to hypoxia. J Appl Physiol (2007) 102(3):972–7. 10.1152/japplphysiol.00956.2006 17341736

[60] Mayock DE Ngai AC Mondares RL Gleason CA. Effects of binge alcohol exposure in the second trimester on intracerebral arteriolar function in third trimester fetal sheep. Brain Res (2008) 1226:111–5. 10.1016/j.brainres.2008.05.077 18640664 PMC2674847

[61] Ngai AC Mondares RL Mayock DE Gleason CA. Fetal alcohol exposure alters cerebrovascular reactivity to vasoactive intestinal peptide in adult sheep. Neonatology (2008) 93(1):45–51. 10.1159/000105524 17630497

[62] Simon KE Mondares RL Born DE Gleason CA. The effects of binge alcohol exposure in the 2nd trimester on the estimated density of cerebral microvessels in near-term fetal sheep. Brain Res (2008) 1231:75–80. 10.1016/j.brainres.2008.06.125 18657528 PMC2583365

[63] Han M Neves AL Serrano M Brinez P Huhta JC Acharya G Effects of alcohol, lithium, and homocysteine on nonmuscle myosin-II in the mouse placenta and human trophoblasts. Am J Obstet Gynecol (2012) 207(2):140 e7–19. 10.1016/j.ajog.2012.05.007 PMC3408570 22704764

[64] Bake S Tingling JD Miranda RC. Ethanol exposure during pregnancy persistently attenuates cranially directed blood flow in the developing fetus: Evidence from ultrasound imaging in a murine second trimester equivalent model. Alcohol Clin Exp Res (2012) 36(5):748–58. 10.1111/j.1530-0277.2011.01676.x 22141380 PMC3297711

[65] Subramanian K Naik VD Sathishkumar K Yallampalli C Saade GR Hankins GD Chronic binge alcohol exposure during pregnancy impairs rat maternal uterine vascular function. Alcohol Clin Exp Res (2014) 38(7):1832–8. 10.1111/acer.12431 24962648 PMC4107157

[66] Li X Gao A Wang Y Chen M Peng J Yan H Alcohol exposure leads to unrecoverable cardiovascular defects along with edema and motor function changes in developing zebrafish larvae. Biol Open (2016) 5(8):1128–33. 10.1242/bio.019497 27422904 PMC5004616

[67] Seleverstov O Tobiasz A Jackson JS Sullivan R Ma D Sullivan JP Maternal alcohol exposure during mid-pregnancy dilates fetal cerebral arteries via endocannabinoid receptors. Alcohol (2017) 61:51–61. 10.1016/j.alcohol.2017.01.014 28554529 PMC5517095

[68] Bake S Gardner R Tingling JD Miranda RC Sohrabji F. Fetal alcohol exposure alters blood flow and neurological responses to transient cerebral ischemia in adult mice. Alcohol Clin Exp Res (2017) 41(1):117–27. 10.1111/acer.13277 27987329 PMC5501092

[69] Lecuyer M Laquerriere A Bekri S Lesueur C Ramdani Y Jegou S PLGF, a placental marker of fetal brain defects after *in utero* alcohol exposure. Acta Neuropathol Commun (2017) 5(1):44. 10.1186/s40478-017-0444-6 28587682 PMC5461764

[70] Bouskila J Palmour RM Bouchard JF Ptito M. Retinal structure and function in monkeys with fetal alcohol exposure. Exp Eye Res (2018) 177:55–64. 10.1016/j.exer.2018.07.027 30071214

[71] Tobiasz AM Duncan JR Bursac Z Sullivan RD Tate DL Dopico AM The effect of prenatal alcohol exposure on fetal growth and cardiovascular parameters in a baboon model of pregnancy. Reprod Sci (2018) 25(7):1116–23. 10.1177/1933719117734317 28982294 PMC6346348

[72] Naik VD Davis-Anderson K Subramanian K Lunde-Young R Nemec MJ Ramadoss J. Mechanisms underlying chronic binge alcohol exposure-induced uterine artery dysfunction in pregnant rat. Alcohol Clin Exp Res (2018) 42(4):682–90. 10.1111/acer.13602 29363778 PMC5880721

[73] Raghunathan R Wu C Singh M Liu CH Miranda RC Larin KV. Evaluating the effects of maternal alcohol consumption on murine fetal brain vasculature using optical coherence tomography. J Biophotonics (2018) 11(5):e201700238. 10.1002/jbio.201700238 29292845 PMC6292438

[74] Bisen S Kakhniashvili D Johnson DL Bukiya AN. Proteomic analysis of baboon cerebral artery reveals potential pathways of damage by prenatal alcohol exposure. Mol Cel Proteomics (2019) 18(2):294–307. 10.1074/mcp.RA118.001047 PMC6356072 30413562

[75] Simakova M Tobiasz A Sullivan RD Bisen S Duncan J Sullivan JP Gestational age-dependent interplay between endocannabinoid receptors and alcohol in fetal cerebral arteries. J Drug Alcohol Res (2019) 8:236068. 10.4303/jdar/236068 31057979 PMC6497414

[76] Cananzi SG Mayhan WG. *In utero* exposure to alcohol impairs reactivity of cerebral arterioles and increases susceptibility of the brain to damage following ischemia/reperfusion in adulthood. Alcohol Clin Exp Res (2019) 43(4):607–16. 10.1111/acer.13979 30748017 PMC6538292

[77] Raghunathan R Liu CH Kouka A Singh M Miranda RC Larin KV. Dose-response analysis of microvasculature changes in the murine fetal brain and the maternal extremities due to prenatal ethanol exposure. J Biomed Opt (2020) 25:126001. 10.1117/1.JBO.25.12.126001 33244919 PMC7689263

[78] Adachi J Mizoi Y Fukunaga T Ogawa Y Ueno Y Imamichi H. Degrees of alcohol intoxication in 117 hospitalized cases. J Stud Alcohol (1991) 52(5):448–53. 10.15288/jsa.1991.52.448 1943100

[79] Hasegawa T Ravens JR Toole JF. Precapillary arteriovenous anastomoses. "Thoroughfare channels" in the brain. Arch Neurol (1967) 16(2):217–24. 10.1001/archneur.1967.00470200105010 4163498

[80] Heymann MA Payne BD Hoffman JI Rudolph AM. Blood flow measurements with radionuclide-labeled particles. Prog Cardiovasc Dis (1977) 20(1):55–79. 10.1016/s0033-0620(77)80005-4 877305

[81] Phoon CK Aristizabal O Turnbull DH. 40 MHz Doppler characterization of umbilical and dorsal aortic blood flow in the early mouse embryo. Ultrasound Med Biol (2000) 26(8):1275–83. 10.1016/s0301-5629(00)00278-7 11120365

[82] Qureshi AI El-Gengaihi A Hussein HM Suri MF Liebeskind DS. Occurence and variability in acute formation of leptomeningeal collaterals in proximal middle cerebral artery occlusion. J Vasc Interv Neurol (2008) 1(3):70–2. 22518225 PMC3317293

[83] Bayraktar S Ipek A Takmaz T Yildiz Tasci Y Gezer MC. Ocular blood flow and choroidal thickness in ocular hypertension. Int Ophthalmol (2022) 42(5):1357–68. 10.1007/s10792-021-02123-2 34822054

[84] Gordon MO Kass MA. What we have learned from the ocular hypertension treatment study. Am J Ophthalmol (2018) 189. 10.1016/j.ajo.2018.02.016 PMC5915899 29501371

[85] Cananzi SG Mayhan WG. *In utero* exposure to alcohol alters reactivity of cerebral arterioles. J Cereb Blood Flow Metab (2019) 39(2):332–41. 10.1177/0271678X17728163 28840777 PMC6365603

[86] Sachdev U Lotze MT. Perpetual change: Autophagy, the endothelium, and response to vascular injury. J Leukoc Biol (2017) 102(2):221–35. 10.1189/jlb.3RU1116-484RR 28626046 PMC6608075

[87] Sarkar DK Gangisetty O Wozniak JR Eckerle JK Georgieff MK Foroud TM Persistent changes in stress-regulatory genes in pregnant women or children exposed prenatally to alcohol. Alcohol Clin Exp Res (2019) 43(9):1887–97. 10.1111/acer.14148 31329297 PMC6722014

[88] Osborn JA Kim CK Yu W Herbert L Weinberg J. Fetal ethanol exposure alters pituitary-adrenal sensitivity to dexamethasone suppression. Psychoneuroendocrinology (1996) 21(2):127–43. 10.1016/0306-4530(95)00037-2 8774058

[89] Gray SP Denton KM Cullen-McEwen L Bertram JF Moritz KM. Prenatal exposure to alcohol reduces nephron number and raises blood pressure in progeny. J Am Soc Nephrol (2010) 21(11):1891–902. 10.1681/ASN.2010040368 20829403 PMC3014004

